# Informed consent in minor and intermediate pediatric elective surgery: results of an in-house questionnaire

**DOI:** 10.3389/fsurg.2023.1194657

**Published:** 2023-05-05

**Authors:** Bianca Stefani, Daniela Codrich, Maria Rita Caputo, Edoardo Guida, Maria-Grazia Scarpa, Alessandro Boscarelli, Jürgen Schleef

**Affiliations:** ^1^ Pediatric Surgery Division, Women's And Children's Health Department, University of Padua, Padua, Italy; ^2^ Department of Pediatric Surgery and Urology, Institute of Maternal and Child Health—IRCCS “Burlo Garofolo”, Trieste, Italy

**Keywords:** informed consent, pediatrics, elective surgery, quality improvement, activity questionnaire

## Abstract

**Background:**

The aim of this study was to evaluate the quality of our surgical informed consent and parents'/guardians' late recall of surgical procedures and risks of elective day surgery after pre-operative interview with surgeons.

**Methods:**

All parents/guardians of patients <18 years of age undergoing minor and intermediate elective procedures from January 15th to September 1st, 2022, were prospectively enrolled in the study. Before discharge, parents/guardians were asked to complete an in-house questionnaire regarding the duration of the consent procedure, duration of the interview, quality of the informative handouts, and their ability to recall the type of disease, type of surgical procedure, and surgical risks.

**Results:**

One hundred and two questionnaires were returned. In all cases, informed consent was obtained between 24 and 72 h prior to surgery. The following responses were collected: 98/102 (96%) parents/guardians reported that the duration of the consent process was adequate; 95/102 (93%) reported that the handouts were fully informative, and 7/102 (7%) reported that they were partially informative regarding explanation of the disease and surgical procedure; regarding complications, 93/102 (91%) perceived the handouts to be fully/partially informative, while 4/102 (4%) perceived the handouts to be poorly/non-informative, and 5/102 (5%) did not provide a response; 94/102 (92%) stated that they remembered the pathology, but only 87/94 (93%) recalled it correctly; 90/102 (88%) stated that they remembered the type of procedure, but only 76/90 (84%) recalled it correctly; and 53/102 (52%) stated that they remembered the surgical risks, but only 20/53 (38%) could recall more than one complication.

**Conclusions:**

Late recall of surgical complications by parents was poor despite the high perceived quality of the surgical risk handouts and medical interview. Implementation of expedient methods may improve overall comprehension and satisfaction of parents/guardians regarding the IC process. Further, more efforts should be made to develop standardized guidelines for an optimal IC process.

## Introduction

Informed consent (IC) is a challenging topic for surgeons in everyday practice. Currently, it is not only necessary to formally document the IC process, but it is also a medico-legal affair (1). Surgical IC consists of four key elements: I. provision of adequate information about the disorder, the nature of the treatment and its probability of success, the risks in-volved, and alternative treatments, if any; II. the patient is deemed competent to make the necessary decisions; III. the patient has full understanding of the information provided; and IV. the patient is given freedom to choose (2, 3). To fulfill these criteria, the physician must provide the patient with a clear description of the surgical procedure, the benefits of the procedure, the relevant risks, and all alternative surgical and/or non-surgical treatments (4). The IC process creates an opportunity for parents and/or legal guardians to ask questions and improve their understanding; in addition, it gives the surgeon an opportunity to gain trust and build a good relationship with the family (5).

The aim of our study was to evaluate the quality of our surgical IC and parental late recall of surgical procedures and risks of minor and intermediate surgical elective procedures after the pre-operative interview with surgeons.

## Methods

Parents and/or legal guardians of patients <18 years of age undergoing elective minor and intermediate surgical procedures (e.g., circumcision, inguinal hernia repair, orchiopexy, varicocelectomy, abdominal wall hernia repair, etc.) from January 15th to September 1st, 2022, were prospectively enrolled in the study. Before the procedure, the parents/guardians took part in an interview of about 20–30 min in which written IC was obtained and a surgeon explained the disease, surgical procedure, and associated risks.

After the surgical procedures and before discharge, the parents/guardians were asked to complete an in-house questionnaire developed by the surgical staff to evaluate the overall quality of IC process and parents'/guardians' late recall of surgical procedures and risks. The questionnaire consisted of 14 questions; some were “yes or no” questions, and some presented multiple answers that were graded from 1 to 4. The first question was about the age of the patient and the second was about the type of hospitalization (day hospital or long-stay hospitalization). The three following questions (from 3 to 6) investigated the way in which the IC was obtained and the quality of the informative handouts. Questions 7 to 9 concerned the ability of the parents to recall the type of disease, type of surgical procedure, and surgical risks. Questions 10 and 11 addressed the parents/guardians of patients >14 years of age to determine whether those patients could understand their disease, the procedure they were undergoing, and whether the parent(s)/guardian(s) would agree to allow the patient to submit the consent. Two more queries addressed foreign families to determine whether the consent was understandable and whether they were given the opportunity to call a cultural mediator. The last question intended to gather suggestions to ameliorate the quality of our surgical IC process. An English version of our in-house questionnaire and a copy of our surgical IC have been enclosed as *Supplementary Materials 1 and 2*. The maximum effort has been made to fulfill all items deemed essential for complete, transparent reporting of qualitative research (SRQR) (6).

## Results

One hundred and two anonymous questionnaires from parents/guardians of different nationalities and socio-cultural settings were returned from January 15th to September 1st, 2022. The main results of our study are reported in Table 1. Ninety-two percent of the participants were hospitalized for less than 24 h. The majority of the children (35%) were 0–5 years old, while 30% were 6–10 years old, 13% were 11–14 years old, and children >14 years of age were 22%. In all cases, IC was obtained between 24 and 72 h prior to surgery from different surgeons. The following responses were collected: 98/102 (96%) parents/guardians reported that the duration of the IC process was adequate; 95/102 (93%) reported that the handouts were fully informative, and 7/102 (7%) reported that they were partially informative regarding explanation of the disease and surgical procedure; regarding complications, 93/102 (91%) perceived the handouts as fully/partially informative, while 4/102 (4%) perceived the handouts as poorly/non-informative, and 5/102 (5%) did not provide a response; 94/102 (92%) stated that they remembered the pathology, but only 87/94 (93%) recalled it correctly. Notably, 90/102 (88%) stated that they remembered the type of procedure, but only 76/90 (84%) recalled it correctly; and 53/102 (52%) stated that they remembered the surgical risks, but only 20/53 (38%) could recall more than one complication. Interestingly, the majority (90%) of parents/guardians of patients >14 years of age who were questioned about their child's understanding of the disease and the procedure declared that the patient understood the procedure fully or partially, while 2/21 (10%) declared that the patient did not fully or partially understand the surgical procedure. One-half of interviewees stated that they would allow patients >14 years of age to submit the consent themselves, whereas the other half disagreed, and one parent did not respond. Regarding the questions addressing foreign families to determine whether the consent was understandable and whether they were given the opportunity to call a cultural mediator, 13 of the families were foreign families, and they all agreed that the con-sent was clear; however, 8/13 stated that they were not given the opportunity to call a cultural mediator.

**Table 1:** A tabulated summary of the most relevant results from our survey.

Question	Answers	*N*° (%)
Years of age @ Surgery	*0–5*	36/102 (35)
	*6–10*	31/102 (30)
	*11–14*	13/102 (13)
	*>14*	22/102 (22)
Duration of IC process	*Adequate*	98/102 (96)
	*Inadequate*	4/102 (4)
Handouts on disease and surgical procedure	*Fully informative*	95/102 (93)
	*Partially informative*	7/102 (7)
Handouts on complications	*Fully/Partially informative*	93/102 (91)
	*Poorly/Non- informative*	4/102 (4)
	*Not answered*	5/102 (5)
Remember the pathology	87/94 (93)	
Remember surgical procedure	76/90 (84)	
Remember surgical risks	20/53 (38)	
Comprehension in patients >14 years of age	*Full/Partial*	19/21 (90)

## Discussion

According to the results of our in-house survey, late recall of surgical complications by parents was poor despite the high perceived quality of the surgical procedure and risk handouts, and the overall medical interview. Interestingly, the language barrier appears to continue to play a crucial role in the whole process.

A well-validated definition of IC exists; however, there is no consensus on the specific in-formation that should be provided to patients regarding a surgical operation (7). Most parents/guardians will have firmly decided to proceed before attending the surgery; how-ever, a minority may develop doubts upon learning about the procedure in more detail during the IC process. If these doubts arise on the day of surgery, the parents/guardians may feel pressured to proceed, as the arrangements have already been made (8). In our study, overall, the parents/guardians agreed with the time spent on the IC process was sufficient. In our hospital, we obtain IC at least 24 h before the surgery, during an interview with the parents/guardians and patients. We usually begin with a brief explanation of the disease, the planned operation, the risks and benefits involved, any alternative treatments, and the risks and benefits of not proceeding with the operation. Obtaining IC at least 24 h before the surgery was effective in our study; the parents/guardians felt less pressured to proceed and thus did not feel like they were acting under duress. Furthermore, the parents/guardians reported that the handouts were informative regarding explanation of disease and surgical procedure. Of note, 84% of the parents/guardians correctly recalled the right procedure, whereas only 38% of the parents/guardians recalled more than one surgical complication. These results are in line with those of other published studies reporting poor overall risk recall (4, 7, 9). An explanation for the poor recall rate of surgical risks may be the phenomenon of cognitive dissonance. Cognitive dissonance occurs when two simultaneous thoughts conflict; for example, the information about operative risks conflicts with the belief that surgery is beneficial. To avoid or reduce post-decisional conflicts, people prefer supportive (consonant) information over opposing (dissonant) information (4, 10). Consequently, during the interview with the surgeon, the parents/guardians may have focused on the benefits instead of the risks. Another explanation may be that the process of surgical IC is difficult to fully understand, and parents/guardians are too distracted or too trusting of their physicians to consider the surgical risks. Of note, the use of a professional translator is preferred for family members who may be unfamiliar with medical conditions or may have personal attitudes that influence the efficacy of the translation (11, 12). We highlighted the importance of using a cultural mediator; however, 8/13 foreign families stated that they were not provided the opportunity to call a cultural mediator, and this may be why the recall of surgical risk in this group was poor (9/14). Language barriers can significantly influence the dynamics of the IC process. Therefore, we must emphasize the use of a cultural mediator to facilitate understanding for foreign families.

Currently, the pediatric literature on this field does not provide strong recommendations for effective and adequate IC process. Many efforts have been made to improve the overall process, but they often were limited to specific aspects (13) and/or the intent to develop standardized guidelines did not ameliorate satisfaction or anxiety of parents/guardians (14, 15). Interestingly, the necessity to use interactive computer-based programs to improve the IC process was already addressed in the past (1).

The results and literature that we have discussed so far can identify areas where crucial gaps in the IC process still persist, especially the areas regarding surgical risks, complications, and cultural and language barriers.

This study had several limitations. The IC was obtained by different surgeons. To provide a more objective perspective, a single surgeon should administer all IC forms and perform all the interviews. Furthermore, our study was conducted during the coronavirus disease 2019 pandemic, when we obtained IC from only one parent/guardian. If both of the parents could participate in the interview and IC process, the recall of risks might have been higher. In addition, it had also a potential bias regarding who filled the questionnaire (respondents vs. non-respondents), lacked a correlation of responses to surgeon IC practices, and lacked information on the parents for assessing generalizability. Future research in related fields should be combined with prospective comparative studies with emergency procedures and more rigorous and reasonable designs.

## Conclusions

In conclusion, physicians should make more effort to understand the factors that may affect the IC process with the aim of better informing patients and parents/guardians of the basic risks before surgery. Implementation of relatively simple, expedient methods, such as additional information (e.g., images, brochures, videos), computer-based programs, allotting time for parental concerns and/or questions, and optimizing the setting in which the IC is obtained, may improve overall comprehension and satisfaction of parents/guardians regarding the IC process. More efforts should be made to further improve these aspects and develop standardized guidelines for an optimal IC process.

## Data Availability

The raw data supporting the conclusions of this article will be made available by the authors, without undue reservation.
