# Supplementary material for: Informed consent in minor and intermediate pediatric elective surgery: results of an in-house questionnaire
Source: Front Surg. 2023 May 5;10:1194657. doi: 10.3389/fsurg.2023.1194657 (PMC10196251; doi:10.3389/fsurg.2023.1194657)
Supplement: Supplementary file 1 [file Datasheet1.pdf]

BURLO
IRCCS Materno Infantile Burlo Garofolo - Dipartimento di Chirurgia
S.C. Chirurgia Pediatrica - Direttore Dott. Jurgen Schleef

QUESTIONNAIRE ABOUT SURGICAL INFORMED CONSENT

Dear parents and/or legal guardians,

The questionnaire we are submitting to you, aims to improve the quality of our pediatric patient care, and in particular the quality of communication of information regarding the surgical procedure your child underwent. Please, take a few minutes of your time to answer some simple questions concerning the quality of the information contained in the informed consent and how it was obtained.

1. How old is your child?

- ☐ 0-5
- ☐ 6-10
- ☐ 11-14
- ☐ >14

2. Which type of hospitalization was planned for your child?

- ☐ Day Hospital
- ☐ Long-term Hospitalization

3. Was the written informed consent obtained at list one day before the surgery?

- ☐ YES
- ☐ NO

4. Did the written informed consent include all the information necessary to explain the disease and the surgical procedure?

- ☐ YES, FULLY
- ☐ YES, PARTIALLY
- ☐ NOT TOTALLY
- ☐ NO

5. Did the written informed consent include all the information necessary to understand surgical risks?

- ☐ YES, FULLY
- ☐ YES, PARTIALLY

Istituto di Ricovero e Cura a
Carattere Scientifico materno infantile
Burlo Garofolo
Ospedale di alta specializzazione e di rilievo
nazionale per la salute della donna e del bambino
34137 Trieste via dell’Istria 65/1 tel.+39.040.3785.314 fax +39.040.3785.537 cf. 00124430323

☐ NOT TOTALLY
☐ NO

6. Do you think that the time spent to inform you about the surgery was enough?

☐ YES, FULLY
☐ YES, PARTIALLY
☐ NOT TOTALLY
☐ NO

7. Actually, can you recall the disease that affects your child?

☐ YES
☐ NO

If yes, can you state it? ....
………………………………………………………………………………

8. Actually, can you recall the type of surgical procedure that your child underwent?

☐ YES
☐ NO

If yes, can you state the type of surgery?..
…………………………………………………………………………………..

9. Actually, can you recall the surgical risks of the surgical procedure your child underwent?
☐ YES
☐ NO

If yes, can you list any?
………
………
………………………………………………………………………….

To fill in only if patient > 14 years

10. Do you think your child understood the information communicated by the medical staff regarding his condition and the surgical procedure?

☐ YES, FULLY
☐ YES, PARTIALLY

- ☐ NOT TOTALLY
- ☐ NO

11. Do you agree letting the patients themself submit the consent beside the signature of the parents and/or legal guardians?

- ☐ YES
- ☐ NO

To fill in only if your native language is different from italian.

12. Did the language barrier affect your comprehension of information about the pathology and surgical procedure?

- ☐ YES, FULLY
- ☐ YES, PARTIALLY
- ☐ NOT TOTALLY
- ☐ NO

13. Did you have the possibility to call a cultural mediator, if you need one?

- ☐ YES
- ☐ NO

14. Do you have any suggestions to improve the quality of our informed consent (images, brochures, more time, etc.)?
……
……
……
……
……
……
……………………………………………………………………..

Thank you for filling out our questionnaire.

The Surgical Staff
