# Supplementary material for: Informed consent in minor and intermediate pediatric elective surgery: results of an in-house questionnaire
Source: Front Surg. 2023 May 5;10:1194657. doi: 10.3389/fsurg.2023.1194657 (PMC10196251; doi:10.3389/fsurg.2023.1194657)
Supplement: Supplementary file 2 [file Datasheet2.pdf]

BURLO

ATTO SANITARIO: ....

TRIESTE, ...

- ☐ S.C. Chirurgia e Urologia pediatrica – Direttore Dott. ~~Waifro Rigamonti~~ JÜRGEN SCHLEEF
- ☐ S.C. Ortopedia e traumatologia – Direttore Dott. Marco Carbone
- ☐ S:C. Oculistica e Strabologia – Direttore Dott. Stefano Pensiero
- ☐ S.C. Odontostomatologia pediatrica – Direttore Dott.ssa ~~Gabriella Clarich~~ MILENA CADENARO
- ☐ S.C. Audiologia e Otorinolaringoiatria – Direttore Dott.ssa Eva Orzan

PARTE A

Io/noi sottoscritto/a/i ....

in qualità di ☐ assistito ☐ madre ☐ padre ☐ genitori ☐ tutore ☐ altro (*specificare*) ....

e responsabile/i della compilazione dei dati sottoindicati:

DATI IDENTIFICATIVI

Dati identificativi dell'assistito

Etichetta

Cognome ....Nome.................................

Nato a...il ....

Residente a ....CAP....................

in Via...

Dati dei genitori o del titolare della potestà genitoriale*:

Nome e cognome dei genitori

Madre Cognome ....Nome...............................

Nato a...il ....

Residente a ....CAP....................

in Via...

Padre Cognome .... Nome..............................

Nato a...il ....

Residente a ....CAP....................

in Via...

Titolare potestà genitoriale

Cognome ....Nome.................................

Nato a...il ....

Residente a ....CAP....................

in Via...

**In caso di minore vi è la necessità del consenso fornito da entrambi i genitori, o da chi, giuridicamente, ne fa le veci. Tale assunto non si modifica anche qualora il minore sia affidato ad uno dei genitori, i quali risultino separati o divorziati, come dagli stessi dichiarato. L'opinione del minore, nella misura in cui lo consente la sua età e il suo grado di maturità, è presa in considerazione.*

**In caso di presenza di uno solo dei due genitori, quest'ultimo, sotto la propria responsabilità, dovrà dichiarare l'assenso del genitore assente, attraverso modello di autocertificazione (Mod 0199)*

**In caso di presenza del solo legale rappresentante va allegata la documentazione che indica i poteri conferiti.*

DICHIARO/DICHIARIAMO

di essere stato/a/i informato/a/i in maniera chiara, completa e comprensibile

dal dott. ____

(se del caso) con il supporto del mediatore culturale Sig. ______________________

Istituto di Ricovero e Cura a
Carattere Scientifico materno infantile
Burlo Garofolo
Ospedale di alta specializzazione e di rilievo
nazionale per la salute della donna e del bambino

• 34137 Trieste • via dell'Istria 65/1 • tel.+39.040.3785.314 • fax +39.040.3785.537 • cf. 00124430323 •

Scansionato con CamScanner

Riguardo gli argomenti seguenti

DIAGNOSI / CONDIZIONI CLINICHE

L'assistito è affetto dalla seguente **PATOLOGIA** ....
....
....
....

ATTO SANITARIO

Per il trattamento della patologia di cui è affetto l'assistito è indicato il seguente intervento chirurgico:
....
....

Breve **DESCRIZIONE DELL'INTERVENTO** proposto: ....
....
....
....
....
....
....
....
....

L'intervento è **ESEGUITO DAI SEGUENTI PROFESSIONISTI:** ....
....
....

Il **SANITARIO DI RIFERIMENTO** è il Dott.: ....

Note: ....
....
....

ESITI PREVEDIBILI (BENEFICI) DEL TRATTAMENTO PROPOSTO

I **BENEFICI** del trattamento sono: ....
....
....
....
....

Gli esiti prevedibili del **MANCATO TRATTAMENTO** sono: ....
....
....

LE EVENTUALE ALTERNATIVE (CON VANTAGGI E RISCHI)

Rispetto all'intervento proposto: ☐ NON esistono alternative

☐ Esistono alternative (*specificare tipologia, rischi e benefici*): ....
....
....
....

RISCHI E COMPLICANZE EVENTUALI DEL TRATTAMENTO

Le **COMPLICANZE POST – OPERATORIE IMMEDIATE** e più comuni di questo intervento sono:

☐ reazioni allergiche legate alla somministrazione dell'anestetico locale

☐ sanguinamento durante e dopo l'intervento ☐ tumefazioni originate dall'edema postoperatorio

☐ ematomi

continua

Scansionato con CamScanner

continua RISCHI E COMPLICANZE EVENTUALI DEL TRATTAMENTO

- ☐ infezioni nella sede dell'intervento
- ☐ apertura della ferita chirurgica
- ☐ ……………………………………………………………………………………
- ☐ ……………………………………………………………………………………

Le COMPLICANZE POST – OPERATORIE TARDIVE specifiche per questo intervento sono:

- ☐ lesioni nervose transitorie o permanenti
- ☐ frattura ossea
- ☐ ……………………………………………………………………………………
- ☐ ……………………………………………………………………………………
- ☐ ……………………………………………………………………………………
- ☐ ……………………………………………………………………………………

In linea generale il periodo necessario per il recupero funzionale è di: ……………………………………
……………………………………………………………………………………

ALTRI PROBLEMI

Eventuali variazioni rispetto alla tecnica operatoria proposta possono essere dettate da condizioni anatomo-patologiche impreviste e non preventivate all'atto della somministrazione della presente informativa.

Possono essere altresì necessarie, in condizioni di particolare e specifica gravità dello stato di salute dell'assistito, manovre di resezione, amputazione, asportazione di organi e/o parti anatomiche.

……………………………………………………………………………………
……………………………………………………………………………………
……………………………………………………………………………………
……………………………………………………………………………………

DISEGNO ILLUSTRATIVO *(se necessario)*

Scansionato con CamScanner

PARTE B

ACQUISIZIONE DEL CONSENSO

DICHIARO/DICHIARIAMO

☐ di essere consapevole (e di assumerne la responsabilità civile e penale) che eventuali omissioni rispetto alle informazioni riferite in anamnesi, siano causa di inadeguate condotte mediche e chirurgiche;

☐ di essere a conoscenza della possibilità di revocare il presente consenso in qualsiasi momento prima del Atto/trattamento sanitario proposto e di:

☐ ACCETTARE ☐ NON ACCETTARE

Liberamente, spontaneamente ed in piena coscienza il mio consenso all'atto sanitario proposto.

Data Firma leggibile dell'assistito/a e/o del/i legale/i rappresentante/i

Altri (specificare) Il Medico (timbro e firma leggibile)

PARTE C

DIRITTO DI REVOCA

DICHIARO/DICHIARIAMO DI VOLERE REVOCARE IL CONSENSO

Data Firma leggibile dell'assistito/a e/o del/i legale/i rappresentante/i

Scansionato con CamScanner
